# Lessons for test and treat in an antiretroviral programme after decentralisation in Uganda: a retrospective analysis of outcomes in public healthcare facilities within the Lablite project

**DOI:** 10.1093/inthealth/ihz090

**Published:** 2019-11-15

**Authors:** S Kiwuwa-Muyingo, G Abongomera, I Mambule, D Senjovu, E Katabira, C Kityo, D M Gibb, D Ford, J Seeley

**Affiliations:** Medical Research Council/Uganda Virus Research Institute and London School of Hygiene and Tropical Medicine, Uganda Research Unit, PO Box 49, Entebbe, Uganda; African Population and Health Research Center, P.O. Box 10787-00100, Kitisuru, Nairobi, Kenya; Joint Clinical Research Centre, PO Box 10005, Kampala, Uganda; University of Zurich, Epidemiology, Biostatistics and Prevention Institute, CH 8001, Zurich, Switzerland; Infectious Diseases Institute, Makerere University, PO Box 22418, Kampala, Uganda; Infectious Diseases Institute, Makerere University, PO Box 22418, Kampala, Uganda; Infectious Diseases Institute, Makerere University, PO Box 22418, Kampala, Uganda; Joint Clinical Research Centre, PO Box 10005, Kampala, Uganda; Medical Research Council, Clinical Trials Unit at University College London, London WC1V 6LH, UK; Medical Research Council, Clinical Trials Unit at University College London, London WC1V 6LH, UK; Medical Research Council/Uganda Virus Research Institute and London School of Hygiene and Tropical Medicine, Uganda Research Unit, PO Box 49, Entebbe, Uganda; Global Health and Development Department, London School of Hygiene & Tropical Medicine, London, WC1E 7HT, UK

**Keywords:** antiretroviral therapy, attrition, decentralisation, human immunodeficiency virus, retention, sub-Saharan Africa

## Abstract

**Background:**

We describe the decentralisation of antiretroviral therapy (ART) alongside Option B+ roll-out in public healthcare facilities in the Lablite project in Uganda. Lessons learned will inform programmes now implementing universal test and treat (UTT).

**Methods:**

Routine data were retrospectively extracted from ART registers between October 2012 and March 2015 for all adults and children initiating ART at two primary care facilities (spokes) and their corresponding district hospitals (hubs) in northern and central Uganda. We describe ART initiation over time and retention and use of Cox models to explore risk factors for attrition due to mortality and loss to follow-up. Results from tracing of patients lost to follow-up were used to correct retention estimates.

**Results:**

Of 2100 ART initiations, 1125 were in the north, including 944 (84%) at the hub and 181 (16%) at the spokes; children comprised 95 (10%) initiations at the hubs and 14 (8%) at the spokes. Corresponding numbers were 642 (66%) at the hub and 333 (34%) at the spokes in the central region (77 [12%] and 22 [7%], respectively, in children). Children <3 y of age comprised the minority of initiations in children at all sites. Twenty-three percent of adult ART initiations at the north hub were Option B+ compared with 45% at the spokes (25% and 65%, respectively, in the central region). Proportions retained in care in the north hub at 6 and 12 mo were 92% (95% CI 90 to 93) and 89% (895% CI 7 to 91), respectively. Corresponding corrected estimates in the north spokes were 87% (95% CI 78 to 93) and 82% (95% CI 72 to 89), respectively. In the central hub, corrected estimates were 84% (95% CI 80 to 87) and 78% (95% CI 74 to 82), and were 89% (95% CI 77.9 to 95.1) and 83% (95% CI 64.1 to 92.9) at the spokes, respectively. Among adults newly initiating ART, being older was independently associated with a lower risk of attrition (adjusted hazard ratio [aHR] 0.93 per 5 y [95% CI 0.88 to 0.97]). Other independent risk factors included initiating with a tenofovir-based regimen vs zidovudine (aHR 0.60 [95% CI 0.46 to 0.77]), year of ART initiation (2013 aHR 1.55 [95% CI 1.21 to 1.97], ≥2014 aHR 1.41 [95% CI 1.06 to 1.87]) vs 2012, hub vs spoke (aHR 0.35 [95% CI 0.29 to 0.43]) and central vs north (aHR 2.28 [95% CI 1.86 to 2.81]). Independently, patient type was associated with retention.

**Conclusions:**

After ART decentralisation, people living with human immunodeficiency virus (HIV) were willing to initiate ART in rural primary care facilities. Retention on ART was variable across facilities and attrition was higher among some groups, including younger adults and women initiating ART during pregnancy/breastfeeding. Interventions to support these groups are required to optimise benefits of expanded access to HIV services under UTT.

## Background

In 2017, the number of adults living with human immunodeficiency virus (HIV) in Uganda was estimated at 1.3 million (5.9%).^1,2^ To attain universal access to antiretroviral therapy (ART), decentralisation of HIV treatment from hospitals to primary care (PC) facilities is necessary. Several studies have also shown that decentralisation of ART services to PC improves access to care and improves retention among children and adults.^3–6^

Since 2012, many sub-Saharan African countries have transitioned from a CD4 cell count threshold of 350 cells/mm^3^ to a count of 500 cells/mm^3^ and adopted Option B+, where all pregnant and breastfeeding women are eligible for ART regardless of their CD4 cell count or WHO stage, in accordance with WHO recommendations to eliminate mother-to-child transmission.^7–9^ Decentralisation of ART provision to lower-level PC health facilities was scaled up in November 2012 in Uganda, alongside the introduction of Option B+. Implementation of these strategies occurred in phases, starting in regions with the highest HIV prevalence. In 2013, under the national ART guidelines, all patients identified with HIV infection and a CD4 count of <500 cells/mm^3^ were eligible for ART, up from a CD4 count of <350 cells/mm^3^ in 2010. In March 2014, the Ministry of Health (MOH) in Uganda effected a change in guidelines indicating a regimen of tenofovir (TDF), lamivudine (3TC) and efavirenz (EFV) for first-line treatment following WHO recommendations. Currently ART is to be provided free of charge through the universal test and treat (UTT) approach for all HIV-positive individuals in Uganda following the recommendation from the WHO.^10^ While efforts to scale up increased ART coverage to 72% by the end of 2017,^1,2^ and the proportion of pregnant women starting ART reached 97%,^11^ a number of individuals living with HIV still do not have access to ART. Challenges for programmes remain regarding retention in care and ART use during postnatal care.^7,12–14^ In Uganda, loss to follow-up rates by 6 mo for Option B+ women varied between 10% and 30% in the period 2010–2016,^14–17^ with similarly high attrition in other African settings.^17^ Lessons learned through decentralisation and Option B+ will inform optimal strategies for UTT, for example, indicators of the quality of HIV care, including routine counselling and testing, and subsequent initiation of ART.

The Lablite project worked with the MOHs in Malawi, Zimbabwe and Uganda to evaluate ART rollout in non-research sites in the three countries. In Uganda, Lablite was present in two districts, Agago in the north and Kalungu in central/southwest Uganda, districts that differed in terms of their catchment populations and settings. Both sites comprised a district hospital (hub) and two PC facilities in the surrounding area (spokes).^9^ We used the staff at the hub to train, mentor and support the spokes and to build confidence of lower-level healthcare staff to initiate and monitor ART. Subsequently patient referrals upwards or downwards were based on the level of expertise and service need.

We aimed to document outcomes, treatment changes, retention and risk factors for attrition due to loss to follow-up and mortality among children and adults newly initiated on ART between 2012 and 2015. We summarise key lessons learned following ART decentralisation after Option B+ and implementation of changes in ART guidelines in Uganda and consider the implications for optimising UTT implementation.

## Methods

### Study setting, design and population

**Figure 1:**
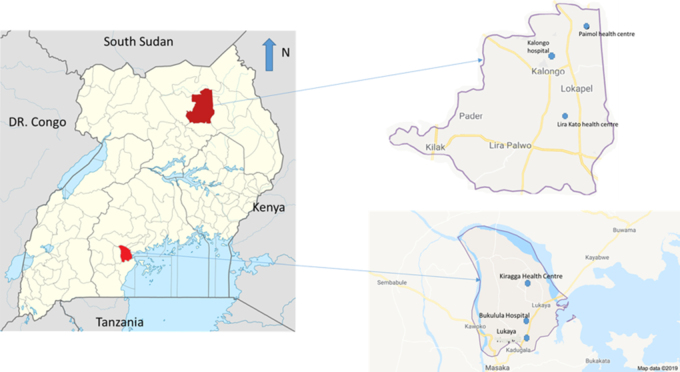
Map of health facilities within the Lablite project in Uganda

Between October 2012 and March 2015 the Uganda MOH implemented Option B+ and decentralisation of ART services to PC. In the Lablite project, we retrospectively collected routine patient-level data for children and adults enrolled in HIV care in two geographical regions, including the district hospital (hub) and two corresponding PC health facilities (spokes). Through Lablite, working with the MOH, we implemented an HIV care task-shifting model from hospital-based physician to primary care nurse in November 2012 as the MOH rolled out Option B+ in PC health facilities. Sites were chosen in consultation with MOH. The Agago district in the north is a post-conflict area that is difficult to reach. It has an HIV prevalence of 8.2% and a catchment of 285 300. The Kalongo hospital (hub) has provided ART since 2005, is owned by the Uganda Roman Catholic church and, through Lablite, was linked to two PC health facilities (spokes), the Paimol and Lira Kato health centres in the district (Figure 1). Prior to decentralisation of ART services, Paimol and Lira Kato had no ART outreach programmes and patients had to take an approximately 56 km round trip to Kalongo hospital (or 76 km to Patongo hospital) for ART.^18^ Option B+ in these PC facilities started in April 2013, followed by general ART provision starting in May 2013. After ART decentralisation, Paimol and Lira Kato provided ART to catchments of 23 940 and 19 215, respectively. The hub or nearby hospitals in the north provided CD4 cell count testing facilities for samples taken at the PC facilities so patients did not have to travel, but there were technical challenges with the CD4 testing machine at the hospital, resulting in testing not always being available.

In June 2014, the Kalongo hospital implemented the increased CD4 threshold for ART initiation and subsequently changed first-line treatment to TDF, 3TC and EFV in July 2014. The MOH implemented these changes in ART national guidelines in a tiered approach (at higher-level facilities first), with training from hospitals to PC. ART initiation and/or regimen provision depended on guidelines and the drug supply chain of implementing partners. The MOH provided onsite training, with the Lablite team, to all HIV care health facility staff through a hub–spoke mentorship training module on new drug regimen dosing, side effects and supply chain management prior to the implementation of changes, resulting in varying implementation dates for health facilities (Table 1).^9^

**Table 1:** Dates by facility for implementation of Option B+, decentralisation and changes in treatment guidelines for patients newly initiated on ART in two district hospitals (hubs) and corresponding primary healthcare units (spokes) between 2013 and 2015

	Central hub (Bukulula)	Central spoke (Lukaya)	Central spoke (Kiragga)	North hub (Kalongo)	North spoke (Paimol)	North spoke (Lira Kato)
Option B+	October Q4 2012	October Q4 2012	Q4 2012	October Q4 2012	April Q2 2013	April Q2 2013
General ART provision	2005	March Q1 2013	March Q2 2013	2005	April Q2 2013	April Q2 2013
Change in CD4 threshold from 350 to 500 cells/mm^3^	Q2 2014	Q2 2014	Q2 2014	March/April Q2 2014	August Q3 2014	September Q3 2014
Regimen change to first-line ART TDF/3TC/EFV	June Q2 2014	July Q2 2014	August Q2 2014	July Q3 2013*	September Q4 2013	September Q4 2013

Q1: first quarter; Q2: second quarter; Q3: third quarter; Q4: fourth quarter.

Tenofovir, lamivudine and efavirenz (TDF/3TC/EFV) first line antiretroviral therapy.

The Kalungu (central) district has an HIV prevalence of 12.5% and a catchment of approximately 44 300.^18^ The district includes truck driver stops along a national highway and fishing villages. ART has been available since 2005 through the health services in a health centre designated as the equivalent of a district hospital (Bukulula). During the Lablite project, the Bukulula facility (hub) was linked to two PC facilities as spokes, Lukaya and Kiragga (Figure 1). Prior to decentralisation, Bukulula provided outreach ART to the Lukaya PC facility (whereby staff from Bukulula visited Lukaya 1 d to provide ART consultations), while another private provider serviced the Kiragga PC facility. Option B+ provision at the PC facilities started in October 2012 (Table 1). The MOH, working with Lablite, started general ART provision at PC facilities in April 2013, with Lukaya and Kiragga providing ART to catchments of approximately 5600 and 9000 individuals, respectively. Changes in CD4 thresholds for ART initiation were implemented in the second quarter of 2014. Bukulula implemented a regimen change to first-line TDF, 3TC and EFV in May 2014, following MOH training, with the change in PC occurring approximately 3 mo later. Samples were collected at the PC facilities and sent to the Bukulula hub for CD4 cell count testing.

### Data collection

Data clerks extracted and entered data into a bespoke database from facility ART registers between October 2012 and March 2015 for all adults and children newly initiating ART. We captured ART register data on sex, age, WHO stage, weight and CD4 cell count at ART initiation and monthly follow-up data for ART use, treatment changes (including interruption), transfers out of the facility, information on pregnancy and breastfeeding, and tuberculosis status. Assessments for WHO disease stage and CD4 cell count were scheduled at the 6 mo visits. In a few cases a 2 mo supply of ART was given at the physician’s discretion if the patient had a history of good adherence. Individuals on ART could send someone else to pick up their drugs; this was more common in the north due to the long distances travelled by clients to reach ART facilities.^19^ In 2014, the MOH introduced an open medical record system electronic database tool at the Bukulula hub. Alongside the paper ART registers, data clerks entered patient data into the electronic tool retrospectively. In Lablite, this resulted in delays in updating the ART register. At the Kalongo hospital, a data clerk extracted data from a pre-existing electronic data system. For all the PC facilities, data clerks collected information from the paper ART registers. We checked data for consistency, raised queries and made changes as necessary with support from health workers and the district data clerks. We obtained additional information from patient cards in response to queries or if data were missing from the ART register.

**Table 2:** Reasons for initiating ART at the hubs and spokes among children, adult men, Option B+ women and women

	Type of patient	Reason for initiating ART	Central hub, n (%)	Central spokes, n (%)	North hub, n (%)	North spokes, n (%)	Total
Group 1	Children newly initiated on ART	<15 y	77 (37.0)	22 (10.6)	95 (45.7)	14 (6.7)	208 (100)
	Adults newly initiated on ART	≥15 y	565 (29.9)	311 (16.4)	849 (44.9)	167 (8.8)	1892 (100)
Group 2	Men in need of ART (% of all adults at facility)	CD4 <350/500 cells/mm^3^ or WHO 3/4	146 (25.8)	28 (9.0)	145 (17.1)	29 (17.4)	348 (16.6)
	Men in need of ART^a^ (% of all adults at facility)	Unknown CD4 or WHO stage	15 (2.7)	4 (1.3)	137 (16.1)	8 (4.8)	164 (7.8)
Group 3	Option B+ women (% of all adults at facility)	Pregnant or breastfeeding	143 (25.3)	202 (65.0)	193 (22.7)	75 (44.9)	613 (29.2)
Group 4	Women in need of ART^b^ (% of all adults at facility)	CD4 <350/500 cells/mm^3^ or WHO 3/4	220 (38.9)	59 (19.0)	147 (17.3)	39 (23.4)	465 (22.1)
	Women in need of ART^a^ (% of all adults at facility)	Unknown CD4 or WHO stage	41 (7.3)	18 (5.8)	227 (26.7)	16 (9.6)	302 (14.4)

a If WHO disease staging 1/2 and unknown CD4 or CD4 >threshold and unknown stage or unknown stage and unknown CD4.

b Women in need of ART for their own health: CD4 <350/500 cells/mm^3^ or WHO stage 3 or 4; Option B+ women, presence of an ANC number, expected delivery date for pregnancy or EID number for breastfeeding in the ART register.

All men were classified as needing ART for their own health. Women were subdivided into Option B+ clients and those starting ART for their own need. All women who started ART at a CD4 below the threshold for initiation by date (Table 1) and/or had WHO stage 3/4 disease were classified as starting ART for their own need. Other women were classified as follows. At the central hub and spokes, we differentiated Option B+ patients from other female patients in the ART register by the presence of an antenatal care (ANC) number, an expected delivery date for pregnancy or an early infant diagnosis (EID) number for breastfeeding (Table 2). We believed these fields were poorly completed (particularly early on), hence, in addition, before June 2014 (when regimen changes were implemented at the central hub), all women who initiated on TDF/3TC/EFV (with CD4 unknown or ≥350 cells/mm^3^) were assumed to be Option B+ women; similar assumptions were made for women starting ART at the central spokes before July 2014 (with CD4 cut-off increased to ≥500 cell/mm^3^ for initiations in June 2014). Once TDF/3TC/EFV was being used as the first-line regimen at a site, we were unable to use regimen to distinguish Option B+ women from other women. Thus we used pregnancy date, EID/ANC number, WHO stage and CD4 count where available, and otherwise assumed women were starting ART for need. In the north, the patient source (captured in the electronic data at the hub) was used to classify Option B+ women: women who were recorded as entering the HIV programme for prevention of mother-to-child transmission (PMTCT) and started ART within 1 mo of entry were assumed to have started Option B+. This did not allow us to capture women starting Option B+ during breastfeeding. At the north spokes, information on the paper ART registers (including regimen, WHO stage, CD4 count and date of initiation) was used similarly to in the central spokes. In the north, WHO staging was often not completed due to a lack of understanding (particularly at the hub); although improvements were made over time, health system challenges still existed, with limited human resources and high turnover of staff.

Health workers recorded data on mortality, if available, from registers, usually informed by peer clients. There were systems in place through a non-governmental organization to trace all clients lost to follow-up in the north hub. We actively tried to trace a sample of all adult patients lost to follow-up at the other facilities that were working with Lablite through either community contacts, peer clients or phone calls, where phone contacts were available and clients had consented. At most, three tracing attempts were made. Tracing outcomes included death, transfer to another ART facility or dropped out of care (i.e. genuinely lost from follow-up).

### Statistical analysis

Data on individuals newly initiating ART during the study period were included; participants already on treatment who transferred in to facilities were excluded. Descriptive analyses of patient profiles included sociodemographic variables using proportions and median (IQR). We present data on children <3 y and 3–14 y of age and adults (≥15 y of age), as guidelines have used these categories for treatment regimens and ART initiation. Adult groups were identified as B+ women, non-B+ women and men. We explored associations between groups and baseline characteristics using χ^2^ tests for categorical variables and rank sum tests for continuous variables.

Attrition was defined as death or loss to follow-up (not seen for >3 mo since the last ART supply). Crude rates for attrition and 95% CIs around the rates are provided. We used Kaplan–Meier methods to estimate retention (1−attrition) in care differentiated by service provision (spoke vs hub). Differences in retention between groups (type of health facility) were assessed using log rank tests. A naive analysis using passively recorded deaths from routine clinic data and loss to follow-up as defined above was conducted initially. Adjusted estimates were obtained based on tracing outcomes. A weight of 1 was assigned to all patients whose outcomes were known prior to tracing, a weight of 0 was assigned to all patients with unknown outcome (either lost to follow-up and not traced or lost to follow-up) and a weight equal to the ratio of all patients lost to follow-up over those lost and sampled with tracing outcomes for patients traced with vital status.^20^ Weights were calculated separately by region and facility level (hub/spoke). Although tracing was attempted at both central spokes, we only received information from one: because the patient characteristics were different at the two spokes, we only made adjustment to estimates from the one spoke with tracing information.

We investigated risk factors for attrition in separate Cox regression analyses for children and adults newly initiated on ART. We used backwards model selection, where we start with all factors of interest, irrespective of univariable analysis. We present univariable and multivariable Cox regression models, including HRs, 95% CIs and p-values. We considered age, sex, year of ART initiation (2013), starting regime and patient type (B+ women, adult men and adult women in need of ART), region and type of facility as potential predictors (note that the CD4 cell count was more likely to be missing among B+ women since it was not required for eligibility). Analyses were performed using Stata version 15.1 (StataCorp, College Station, TX, USA).

## Results

**Figure 2:**
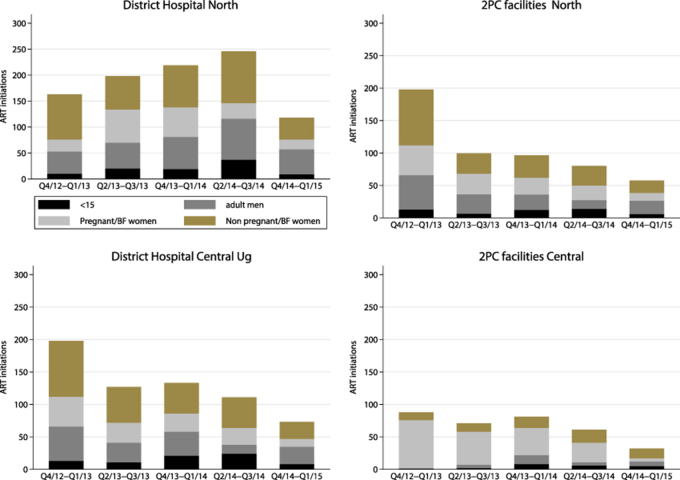
Numbers of patients newly initiated on ART by patient status and type of facility (hospital and Primary care (spokes) health facilities

Baseline characteristics of participants in the two hubs and corresponding spokes are presented in Table 3. There were 2100 ART initiations at the two hubs and four spokes. Of these, 1892 (90%) were in adults and 208 (10%) were in children. In northern Uganda there were 1125 ART initiations, including 944 (84%) at the hub and 181 (16%) at the spokes. At the north hub, 95 (10%) initiations were in children; in comparison, 14 (8%) initiations across both spokes were in children (Figure 2). In central Uganda there were 975 ART initiations, 642 (66%) at the hub and 333 (34%) at the spokes. At the central hub, 77 (12%) initiations were in children, compared with 22 (6%) at the two spokes. Figure 2 shows changes in uptake of ART after Option B+ and the decentralisation of ART from the hub to spokes in 6 mo periods. The proportion of ART initiations in children 0–14 y of age (of all ART initiations) increased from 6% at 0–6 mo after Option B+ to 9% at 6–12 mo, 8% at 12–18 mo and 14% at 18–24 mo in the north. Similarly, central ART initiations in children increased from 5% at 0–6 mo to 7% at 6–12 mo, 14% at 12–18 mo and 17% at 18–24 mo. We observed a decline in ART initiations at both hubs following decentralisation, from the second quarter of 2014 in the north and the second quarter of 2013 in the central region. At the spokes there was a steady increase in patient numbers enrolled over time, including patients in need of ART.

**Table 3:** Baseline characteristics of patients initiating ART in two district hubs and spokes in Uganda

Characteristics	Central				North			
	Hub	% of total or of subgroup	Spokes	% of total or of subgroup	Hub	% of total or of subgroup	Spokes	% of total or subgroup
Children								
Number enrolled	77	77	22	22	95	10	14	8
Age (years)								
<3	16	67	8	33	32	89	4	11
3–14	61	81	14	19	63	86	10	14
Adult males								
Number enrolled	161	83	32	17	282	88	37	12
Age (y), median (IQR)	37 (29–46)		40 (31–51)		35 (30–41)			35 (30–45)
WHO stage								
Stage 1/2	129	80	19	60	107	38	15	41
Stage 3/4	31	19	11	34	11	4	22	59
Missing	1	1	2	6	164	58	0	0
CD4								
Number with CD4 cell counts	148	92	24	75	179	63	20	54
Median (IQR)	245 (135–338)		222 (153–288)		287 (181–440)		274 (179–380)	
Option B+ adult females								
Number enrolled	143	41	202	59	193	72	75	28
Age (y), median (IQR)	27 (22–32)		25 (22–29)		26 (21–31)		27 (21–30)	
WHO stage								
Stage 1/2	142	99	199	98	161	83	75	100
Stage 3/4	0	0	0	0	0	0	0	0
Missing	1	1	3	2	32	17	0	0
CD4								
Number with CD4 cell counts	85	59	69	34	67	35	7	9
Median (IQR)	546 (440–777)		508 (426–733)		614 (485–733)		659 (440–755)	
Women in need of ART								
Number enrolled	261	77	77	23	374	87	55	13
Age (y), median (IQR)	30 (24–40)		29 (24–36)		33 (26–42)		30 (24–40)	
WHO stage								
Stage 1/2	231	88	61	79	106	28	30	55
Stage 3/4	29	11	13	17	12	3	24	44
Missing	1	1	3	4	256	69	1	2
Number with CD4 cell counts	231	89	58	75	203	54	25	45
Median (IQR)	266 (172–332)		242 (191–333)		322 (230–450)			290 (137–356)

### Pre-ART decentralisation characteristics

The median age at ART initiation in children <15 y of age was 4.1 y (IQR 1.7–7.6) in the north vs 4.9 y (IQR 2–7) in the central region. As shown in Table 3, overall there were no significant differences in the proportion of children <3 y vs 3–15 y of age between the hub and spokes (p=0.5). Within the region there were no significant differences in the ages of children initiating ART between the hub and spokes: 4.6 y (IQR 1.7–7.9) vs 4.0 y (IQR 1.6–7) (p=0.4) in the north and 4.9 y (IQR 2–7) vs 3.0 y (IQR 1.4–8) (p=0.4) in the central region, respectively. Of all ART initiations in children <15 y of age, children <3 y of age were in the minority at all sites, 33% in the north and 26% in the central region.

The median age among adults ≥15 y of age newly initiating ART in the north was 31 y (IQR 25–39) vs 29 y (IQR 24–37) in the central region. Within regions there were differences in the ages of adults initiating ART between the hub and spokes: 31 y (IQR 26–39) vs 29 y (IQR 24–36) (p=0.001) in the north and 30 y (IQR 24–40) vs 26 y (IQR 23–32) (p<0.001) in the central region. Overall, Option B+ patients were younger than patients in need of ART: in the north the median age was 26 y (IQR 21–30) among Option B+ women vs 34 y (IQR 27–50) in patients in need of ART (p<0.001) and in the central region the median age was 25 y (IQR 22–30) among Option B+ women vs 32 y (IQR 25–42) in patients in need of ART (p<0.001).

### Reasons for initiating ART

Men or women in need of treatment either had a CD4 count <350/<500 or WHO disease stage 3 or 4, or had tuberculosis. The three groups of patients considered were adult men (all in need of ART for their own health), Option B+ women and women in need of ART for their own health. Approximately one-quarter (23%) of adult ART initiations at the north hub were Option B+ compared with 45% at the spokes (p<0.001), with higher proportions of men in need of ART (33% at the hub vs 22% at the spokes) and women in need of ART at the hub (44% at the hub vs 33% at the spokes) (Table 2). In the central region, 25% of adult initiations were Option B+ at the hub compared with 65% at the spokes (p<0.001); a higher proportion of men in need of ART initiated at the hub than at the spokes (29% vs 10%; p<0.001), similar to the women in need of ART (46% vs 25% at the hub and spokes, respectively).

The WHO staging was frequently missing in the north, particularly at the hub (Table 3). A high proportion of adult men initiating ART (approximately 55%), females initiating ART for their own health (69%) and Option B+ women (17%) at the hub had no staging information. Corresponding proportions at the spokes in the north were only seen in women accessing ART through Option B+. The WHO staging at the hub and spokes was similar in the central region: 89% and 90% were WHO stage 1/2 and 11% and 8% were WHO stage 3/4, and there were very few patients with missing staging.

Overall, two-thirds of adult patients initiating ART had a baseline CD4 cell count, with a median of 315 cells/mm^3^ (IQR 207–462). Option B+ simplified ART initiation among pregnant women, as they did not require CD4 cell count testing. Baseline CD4 cell counts were similar within regions. In the north, CD4 testing was done intermittently at both the hub and the spokes, and baseline CD4 was similar in men and women in need of ART: 179/282 (63%) adult men had a CD4 count at the hub, with a median 287 cells/mm^3^ (IQR 181–440) compared with 20/37 (54%) men with a median 274 cells/mm^3^ (IQR 179–380) at the spokes. Corresponding estimates among women in need of ART were 10/27 (37%) with a median 289 cells/mm^3^ (IQR 131–324) and 25/55 (45%) with a median 290 cells/mm^3^ (IQR 137–356) at the hub and spokes, respectively. In the central region, the median CD4 cell counts were similar among men and women in need of ART: the median CD4 cell count among 148/161 (91%) adult men in need of ART was 245 cells/mm^3^ (IQR 135–338) at the hub compared with a median CD4 count in 24/32 (75%) men at the spokes of 222 cells/mm^3^ (IQR 153–288). The median CD4 count for 231/261 (89%) women in need of ART was 266 cells/mm^3^ (IQR 172–332) at the hub and for 58/77 (75%) women it was 242 cells/mm^3^ (IQR 191–333) at the spokes. The majority of women initiating ART for Option B+ did not have a CD4 count (in the north, 65% at the hub and 91% at the spokes; in the central region, 41% at the hub and 66% at the spokes); among all Option B+ women with a pre-ART CD4 count, the median was 562 cells/mm^3^ (IQR 445–744).

### First-line and second-line ART in children

The majority (71%) of children <15 y of age were started on zidovudine (AZT), 3TC and nevirapine (NVP); 10% on abacivir (ABC), 3TC and EFV; 7% on ABC, 3TC and NVP; 4% on AZT, 3TC and EFV; 3% on TDF, 3TC and EFV; 3% on stavudine (d4T), 3TC and NVP; and approximately 1% on other regimens. From the last quarter (Q4) of 2013 to the first quarter (Q1) of 2014 there was a shift from AZT to regimens with ABC in the central region, but in the north, AZT/3TC/NVP (Table 4) was maintained.

**Table 4:** Initial ART regimens in patients newly initiated on ART before and after 2014 following decentralisation and changes in treatment guidelines

First-line ART in children <15 y of age	Before 2014				After 2014			
	Central hub	Central spokes	North hub	North spokes	Central hub	Central spokes	North hub	North spokes
TDF/3TC/EFV or NVP	1 (3)	–	2 (5)	–	2 (4)	1 (6)	4 (8)	–
AZT/3TC/EFV or NVP	25 (80.7)	2 (50)	37(94.8)	3(100)	20 (43.4)	8 (44)	51 (91.1)	10 (91)
D4T/3TC/EFV or NVP	5 (16)	1 (25)	–	–	–	–	–	–
ABC/3TC/EFV or NVP	–	1 (25)	–	1 (11)	23 (49.9)	9 (50)	–	1 (9)
ABC-3TC-LPV	–	–	–	–	1 (2)	–	–	–
First line ART in adults, non-Option B+ patients	Central hub	Central spokes	North hub	North spokes	Central hub	Central spokes	North hub	North spokes
TDF/3TC/EFV or NVP	70 (26.4)	28 (54.9)	316 (96)	14 (77.8)	131 (83.4)	48 (82.3)	324 (98.3)	52 (70.3)
AZT/3TC/EFV or NVP	194 (73.1)	22 (43.1)	9 (3)	4 (22.2)	26 (16.5)	10 (17.2)	4 (1)	22 (29.7)
D4T/3TC/EFV or NVP	–	–	–	–	–		–	
ABC/3TC/EFV or NVP	1 (1)	–	–	–	–		–	
ABC/3TC/LPV	–	–	–	–	–		–	
AZT/3TC/ATV/r	–	1 (2)	–	–	–		–	
First line ART in adults, Option B+ women	Central hub	Central spokes	North hub	North spokes	Central hub	Central spokes	North hub	North spokes
TDF/3TC/EFV or NVP	88 (99)	146 (100)	116 (99)	50 (100)	53 (98.2)	56 (100)	75 (99)	25 (100)
AZT/3TC/EFV or NVP	1 (1)		1 (1)		1 (1.9)		1 (1)	
D4T/3TC/EFV or NVP	–	–	–	–	–	–	–	–
ABC/3TC/EFV or NVP	–	–	–	–	–	–	–	–
ABC/3TC/LPV	–	–	–	–	–	–	–	–
AZT/3TC/ATV/r	–	–	–	–	–	–	–	–

### First-line and second-line ART in adults

Overall, the majority of adults newly initiated on ART started a TDF-based regimen (Table 4). In the north prior to 2014, 96% of adult men and women in need of ART at the hub initiated ART with a TDF-based regimen vs 78% at the spokes (based on only 18 patients); the remaining patients initiated an AZT-based regimen. Prescribing practice remained similar after 2014.

The use of AZT-based ART was higher in the central region prior to 2014, with only 26% of non-Option B+ patients at the hub and 54% at the spokes initiating with a TDF-based regimen. In the central region since 2014, 83% initiated with a TDF-based regimen at the hub and 82% at the spokes, with a shift from AZT to TDF. All but four Option B+ women started a TDF-based regimen.

### Treatment, attrition and retention on ART in children

The median follow-up of 109 children in the north was 11 mo (IQR 7–17) and 12% either died (3) or were lost to follow-up (10). Of the 99 children who started ART in the central region, the median follow-up was 6 mo (IQR 3–12) and 25% either died (1) or were lost to follow-up (25).

In the north, the median follow-up at the hub and spokes was 11 mo (IQR 8–18) and 6 mo (IQR 2–12), respectively, compared with 9 mo (IQR 4–12) and 4 mo (IQR 0–12) in the central region, respectively. Over the follow-up period there was one drug substitution among children in the north and 11 (11%) drug substitutions in the central region (10 at the hub). Over the 12 mo of follow-up, one switch to a second-line ART occurred in the central region.

The overall attrition rate in children was higher in the central region (3.0/100 person-years [95% CI 2.03 to 4.45]) than in the north (1.0/100 person-years [95% CI 0.60 to 1.77]) (p=0.002). Overall attrition rates were lower at the hub than the spokes (1.4/100 person-years [95% CI 0.94 to 2.03] vs 5.6/100 person-years [95% CI 3.19 to 9.90]; p=0.001). Overall attrition rates were also lower in older children starting ART (ages 3–15 y) than in children <3 y of age (1.2/100 person-years [95% CI 0.79 to1.94] vs 3.4/100 person-years [95% CI 2.17 to 5.36]; p=0.001). The age effect was observed in both regions, although it only reached statistical significance in the central region: in the north, attrition rates were 0.7/100 person-years (95% CI 0.31 to 1.52) in children 3–15 y vs 1.8/100 person-years (95% CI 0.87 to 3.82) in children <3 y (p=0.1). Corresponding rates in the central region were 2.0/100 person-years (95% CI 1.14 to 3.40) and 7.0/100 person-years (95% CI 3.95 to 12.25) (p=0.01).

Although data suggested higher attrition at the spokes, with limited data in the north, the difference in attrition rates between the hub and spokes was non-significant: 0.9/100 person-years (95% CI 0.46 to 1.60) vs 3.0/100 person-years (95% CI 0.95 to 9.16), respectively (p=0.1). In the central region, attrition rates were 2.2/100 person-years (95% CI 1.36 to 3.62) at the hub vs 8.0/100 person-years at the spokes (95% CI 4.18 to 15.44) (p=0.01).

**Figure 3:**
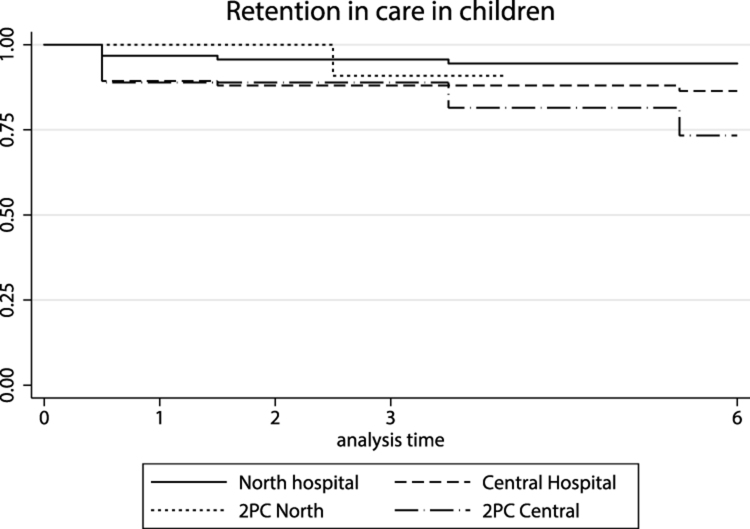
Retention in care in Children by time since ART initiation and type of health facility

Overall retention was 92% (95% CI 86 to 97) and 89% (95% CI 81 to 94) at 6 and 12 mo, respectively, in the north compared with 85% (95% CI 75 to 91) and 75% (95% CI 63 to 83) at 6 and 12 mo, respectively, in the central region (Figure 3).

### Treatment, attrition and retention in care in adults

The median follow-up in the north among 1016 adults newly initiated on ART was 11 mo (IQR 5–19) and 168/1016 (16%) either died (n=26) or were lost to follow-up (n=142). Of 876 adults initiated on ART in the central region, the median follow-up was 6 mo (IQR 2–12) and 392/876 (45%) either died (n=11) or were lost to follow-up (n=381). ART substitution rates among adults were low (0.75/100 person-years in the north over the first 12 mo vs 2.71/100 person-years in the central region (p<0.001). There were 27 (3.2%) first-line substitutions recorded at the hub in the north (17/27 from NVP to EFV) compared with 7 (4.2%) substitutions at the spokes (4/7 from NVP to EFV) (difference in proportion of substitutions between hub and spokes, p=0.5). There were 39 (6.9%) substitutions at the hub in the central region: 16/39 were from AZT/3TC/NVP to AZT/3TC/EFV and 15/39 from AZT/3TC/NVP (n=9) or TF/3TC/EF (n=6) to TDF/3TC/NVP, compared with 4 (1.3%) substitutions at the spokes (3/4 either AZT/3TC/NVP (n=2) or TF/3TC/NVP (n=1) to TDF/3TC/EFV) (difference in proportion of substitutions between hub and spokes, p<0.001). The proportion of patients who switched to second-line treatment was low (<2%): 12 (1.4%) switches to second-line treatment at the hub in the north compared with 11 (1.9%) in the central hub. Second-line regimens included lopinavir/ritonavir (LPV/r) or atazanavir/ritonavir (ATV/r). We only found two switches at the central spokes and none at the spokes in the north.

**Figure 4:**
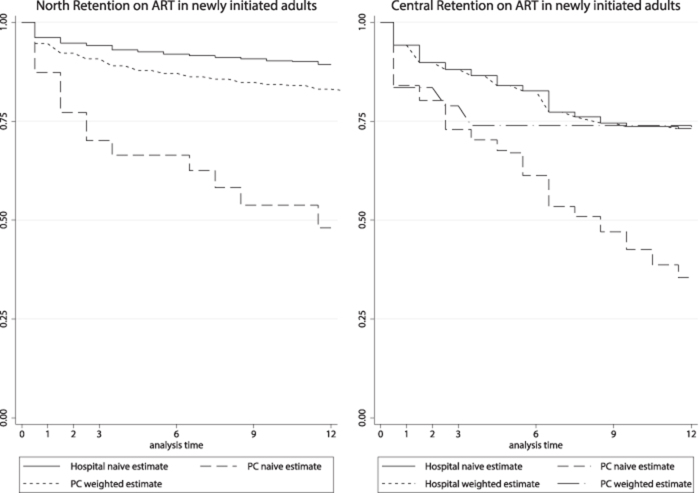
Retention in care in adults by time since ART initiation and type of health facility

Overall crude attrition rates were lower at the hubs (1.7/100 person-years [95% CI 1.54 to 1.94]) compared with the spokes (8.5/100 person-years [95% CI 7.51 to 9.53]). Overall crude attrition rates in adults were higher in the central region than in the north (5.1/100 person-years (95% CI 4.58 to 5.58) vs 1.4/100 person-years (95% CI 1.20 to 1.62]; p<0.001). In a naïve analysis at 12 mo, estimated retention in care was 83% (95% CI 80 to 85) in the north compared with 57% (95% CI 53 to 61) in the central region (Figure 4). In a naïve analysis at 6 mo, overall retention in care at the hub was 86% (95% CI 84 to 87) vs 62% (95% CI 57 to 66) at the spokes. In a naïve analysis at 12 mo, overall retention in care at the hub was 80% (95% CI 78 to 82) vs 43% (95% CI 38 to 48) at the spokes. Proportions retained in care at the hub in the north at 6 and 12 mo were 92% (95% CI 90 to 93) and 89% (95% CI 87 to –91), respectively, compared with 76% (95% CI 73 to 80) and 65% (95% CI 61 to 69) at the central hub. In a naïve analysis at 6 mo, retention in care at the spokes was 58% (95% CI 49 to 65) in the north and 64% (95% CI 58 to 69) in the central region. At 6 mo after decentralisation the estimated retention at the hub among Option B+ women was 89% (95% CI 84 to 93) in the north and 69% (95% CI 61 to 76) in the central region. Option B+ retention at the spokes at 6 mo was lower than at the hubs (53% [95% CI 41 to 64] in the north and 59% [95% CI 52 to 66]) in the central region. Estimated retention among adult men at 6 mo was 65% (95% CI 44 to 79) and 62% (95% CI 40 to 77) in the north and central regions, respectively, and the corresponding proportions among women in need of ART were 63% (95% CI 47 to 76) and 77% (95% CI 65 to 86) respectively.

**Table 5:** Tracing and outcomes for adults in care at the central health facilities and north spokes

				Tracing outcomes				
Health facility	All patients	Patients lost to follow-up	Patients lost to follow-up; tracing attempted	Patients traced with vital status ascertained	Death	Self-transferred to alternative health facility	Dropped out of care (1 attempt to trace)	Dropped out of care (>1 attempt to trace)
Central hub	642	207	207	127	1	29	39	58
Central spoke (Kiragga)^a^	98	45	45	14	0	6	3	5
North spokes^b^ Lira Kato Paimol	99 82	41 37	41 37	8 7	0 0	0 0	8 7	0 0

a Tracing information available for one central spoke.

b Tracing was not done for patients at the north hub because no mechanism was in place for tracing lost patients.

### Tracing outcomes in adults

Tracing was implemented routinely at the north hub but not at the north spokes. Of the 207 patients lost to follow-up at the central hub, 127 were successfully traced and outcomes ascertained (1 died, 29 self-transferred to another ART facility, 97 dropped out of care) (Table 5). Patients whose outcome was ascertained through tracing at the central hub were similar in age (p=0.2) and CD4 cell count (p=0.6) to those for whom outcomes could not be ascertained. Corrected estimates for retention, incorporating tracing outcomes (probability weight of 207/127), at the central hub were 84% (95% CI 80 to 87) at 6 mo and 78% (95% CI 74 to 82) at 12 mo. At the central spokes, corrected estimates at 6 and 12 mo were 89% (95% CI 77.9 to 95.1) and 83% (95% CI 64.1 to 92.9), with a probability weight of 45/14 used for the first spoke and no correction made at the second spoke, where tracing information was not available. At the spokes in the north, proportions in care at 6 and 12 mo in naïve analysis were 58% (95% CI 49 to 65) and 46% (95% CI 37 to 54) and corrected estimates (with probability weights 37/7 at the first spoke and 41/8 at the second spoke) were 87% (95% CI 78.1 to 92.8) and 82% (95% CI 71.6 to 89.2). In the central region, stigma and undocumented transfers were more frequently cited as reasons for dropout, whereas in the north, anecdotal reasons for dropping out of care were given as male involvement (where guidelines encourage pregnant women to come with their partners during the first ANC visit) and stigma.

### Risk factors for attrition from care after ART initiation in children and adults

Table 6 shows the univariable and multivariable results of the Cox proportional hazards model. In children, being >3–15 y of age vs <3 y was independently associated with a lower risk of attrition (aHR 0.49 [95% CI 0.25 to 0.95]), as was initiation after 2013 when the first-line regimen change was implemented (aHR 0.37 [95% CI 0.17 to 0.83]). There was also a lower risk of attrition at the hubs (aHR 0.30 [95% CI 0.15 to 0.63]) compared with the spokes and a higher risk in the central region compared with the north (aHR 2.65 [95% CI 1.33 to 5.29]). Among adult men and women newly initiating ART, being older was independently associated with a lower risk of attrition (aHR 0.93 per 5 y [95% CI 0.88 to 0.97]). Other independent risk factors included year of ART initiation (2013 aHR 1.55 [95% CI 1.21 to 1.97), ≥2014 aHR 1.41 [95% CI 1.06 to 1.87] vs 2012), a lower risk at the hubs vs the spokes (aHR 0.35 [95% CI 0.29 to 0.43]), a lower risk on a TDF-based regimen vs AZT (aHR 0.60 [95% CI 0.46 to 0.77]) and a higher risk of attrition in the central region compared with the north (aHR 2.28 [95% CI 1.86 to 2.81]). After adjustment for age, year of ART initiation, drug regimen, type of facility and region, a lower risk was seen in adult men (aHR 0.80 [95% CI 0.60 to 1.06]) and women initiated on ART for their own health (aHR 0.76 [95% CI 0.60 to 0.96]) than in Option B+ women (p=0.07). We further assessed factors using weighted data in adults (correcting for loss to follow-up) and results were qualitatively similar (data not shown).

**Table 6:** Hazard ratios from Cox proportional model for attrition (loss to follow-up and mortality) on ART in children and adults starting ART in hub/spokes after ART decentralisation

	Children					Adults			
	Univariable analysis, crude HR (95% CI)	p-Value	Adjusted HR^a^ (95% CI)	p-Value		Univariable analysis, crude HR (95% CI)	p-Value	Adjusted HR^a^ (95% CI)	p-Value
Factor									
Age (y), 3–15 vs <3	0.34 (0.17 to 0.68)	0.003	0.49 (0.25 to 0.95)	0.04	Age per 5-y increase	0.89 (0.85 to 0.93)	<0.001	0.93 (0.89 to 0.95)	0.003
Sex, male vs female	0.70 (0.36 to 1.36)	0.3			Sex, male vs female	0.66 (0.54 to 0.81)	<0.001		
Hub vs spokes	0.27 (0.14 to 0.55)	<0.001	0.30 (0.15 to 0.63)	0.001	Hub vs spokes	0.25 (0.21 to 0.30)	<0.001	0.35 (0.29 to 0.43)	<0.001
Central vs north region	2.87 (1.46 to 5.64)	0.002	2.65 (1.33 to 5.29)	0.01	Central vs north region	3.30 (2.76 to 3.96)	<0.001	2.28 (1.86 to 2.81)	<0.001
ART initiated after 2013	0.34 (0.16 to 0.73)	0.01	0.37 (0.17 to 0.83)	0.02	Year of ART initiation				
					2012	1	<0.001	1	0.003
					2013	1.26 (1.00 to 1.59)		1.55 (1.21 to 1.97)	
					2014	0.82 (0.63 to 1.07)		1.42 (1.06 to 1.89)	
Drug regimen^b^, ABC vs other (AZT-based regimen)	0.46 (0.11 to 1.95)	0.3			Drug regimen^c^, TDF-based regimen vs AZT-based regimen)	0.60 (0.50 to 0.73)	<0.001	0.60 (0.46 to 0.77)	<0.001
					Option B+ women	1	<0.001	1	0.07
					Men	0.58 (0.46 to 0.72)		0.80 (0.60 to 1.06)	
					Women in need of ART	0.64 (0.54 to 0.77)		0.76 (0.60 to 0.96)	

a Gender not adjusted due to collinearity with patient type.

b ABC-based regimen (ABC/3TC/EFV or ABC/3TC/NVP) and one on ABC/3TC/LPV vs AZT-based regime (AZT/3TC/NVP) or a few on TDF/3TC/EFV/NVP.

c TDF-based regimen (TDF/3TC/EFV or TDF/3TC/NVP) vs AZT-based regimen (AZT/3TC/EFV or AZT/3TC/NVP) and one on AZT/3TC/ATV/r.

## Discussion

We evaluated the rollout of ART in public healthcare facilities in two district hubs and two linked spokes in two districts within the Lablite implementation project in Uganda. After decentralisation we observed an increase in patients enrolled at the spokes in each region over time.

Estimated retention at 6 mo in children was >80% at the hubs and fewer children were seen at the spokes. We observed retention rates in adult general ART similar to those in a previous study in Tanzania that reported 81% retention at 12 mo in public health facilities.^21^ At 6 mo after decentralisation, estimated retention among Option B+ women was 69% in the central region and 89% in the north, compared with 91% in Zimbabwe and 79% in Malawi.^9^ We found that spokes newly initiating ART had significantly lower retention for women initiating through Option B+. Possible explanations for the difference in retention included undocumented or silent transfers, which were also linked to stigma and disclosure. Research in similar settings has shown that lower retention rates in Option B+ women compared with adults could be linked to resistance to start ART and disclosure.^22,23^ In an analysis of African regions of the International epidemiology Databases to Evaluate AIDS (IeDEA), higher retention at 12 mo was associated with a CD4 cell count <350 cells/mm^3^.^24^ Of note, even within UTT, pregnant women lost early in care are at higher risk for mother-to-child transmission. Recent findings show that even within a test and treat programme, many pregnant women did not remain in care. Therefore, facilitating strategies to improve retention remains important.^25^

Retention on ART was very variable across facilities; although it appeared significantly better at the hubs compared with the spokes, we had limited information on losses to follow-up at the spokes through tracing. There were undocumented efforts to improve retention, such as peer mentors at the central hub, and this may have improved retention at the hub and slowly translated into improvements at the spokes. The north included the hard-to-reach population and those who were vulnerable due to conflict, but mechanisms in place to support this population were successful. Although the spokes received monitoring and training support from the district hubs, support was sporadic due to staffing and logistical constraints. Mechanisms to adapt to the increasing number of patients at the spokes, including data collection and monitoring, could have compromised services and quality of care. We found through tracing that loss to follow-up consisted of substantial undocumented transfers for at least one-third of patients, as has been observed in other ART programmes in Uganda and East Africa.^26,27^ Similar to studies in Ethiopia, Lesotho and Malawi, attrition at treatment sites was mainly due to loss to follow-up.^9,28,29^

Importantly, in ART programmes that have been in existence for a longer time, poor retention could be due to undocumented transfers.^13,14,30,31^ Of note, at least 50% of losses to follow-up were silent transfers, which could lead to considerable underestimation of retention. In a meta-analysis by Zurcher et al.,^30^ proportions of patients successfully traced (of those lost to follow-up) ranged from 20 to 100%, with 34% of those successfully traced having died and 23.9% having transferred care; the latter tended to be common in facilities that had provided ART for longer. In our study, both the central and north hubs had provided ART since 2005. At the central hub, we found that 29% of those traced had genuinely transferred care.^9,21–25^

Regional differences in retention between the north and central regions could have been partly due to differences in study setting. Observed differences could be due to more comprehensive follow-up in the north hub (with routine tracing of patients lost to follow-up) compared with the central hub, where any routine tracing was largely restricted to PMTCT clients. The central catchment population at the spokes was comprised of the most at-risk population, including truck drivers, fishermen and commercial sex workers, with a high population HIV prevalence of 18%. Retention at the north hub was comparable to retention seen in Malawi and Ethiopia.^4–8^ Some of the differences across countries may be due to varying definitions of loss to follow-up and more comprehensive follow-up on the Uganda ART registers than in Malawi (where follow-ups are not recorded on the ART register). In a large cohort of children on ART in Malawi, the estimate of loss to follow-up among paediatric HIV patients was 23% at 1 y on treatment.^32^ While these findings are comparable to our proportions in children, knowledge of paediatric treatment outcomes after ART decentralisation in sub-Saharan Africa remains low.^6,32^ This study not only highlights sociodemographic features of healthcare, but examines potential effects of programme characteristics that may affect retention.

Transitions to second-line treatments up to 2 y were comparable to other countries such as Malawi and Ethiopia^8,28^ and remained low (<10%), similar to another research setting in Uganda. Data from 16 African countries documented a switch rate of 7.9% to second-line treatments after 5 y.^34^ A large, successful (close to achieving the 90-90-90 target) ART programme in Rwanda reported that approximately 4% of all ART patients ≥15 y of age had switched to second-line treatment over a decade (2007–2016).^35^ As ART becomes available to all HIV-infected individuals, viral suppression remains the key measure of treatment success. The goal of monitoring ART to maximise first-line treatment options remains important. In 2015, viral load monitoring started under the national drug surveillance and monitoring programme and was only collected in a few patients at the time.

Our findings on younger age as a risk factor for attrition reinforce findings from many other studies in sub-Saharan Africa.^15,21,36^ While CD4 cell count was also a risk factor in the latter studies, this was not seen in our study, as a large number (99%) of Option B+ patients at the spokes in the north and 66% in the central region had no CD4 cell count measurements. A higher attrition rate observed in Option B+ women (with a CD4 cell count >350 cells/mm^3^ and later >500 cells/mm^3^) is indicative of the impact of CD4 cell count.^15^ A higher proportion (55%) of newly initiated adult men and women in need of ART had CD4 cell counts done at the hub than at the spokes (26%). Suboptimal retention is associated with poor viral suppression, increased switching and subsequent mortality. A study in Zimbabwe showed that point-of-care testing for women and CD4 count-specific counselling was associated with retention.^37,38^ It is plausible that knowledge of CD4 cell count or viral load may enhance patient adherence to ART and knowledge of patient health status may sustain patients in care and improve health outcomes. Associations of retention with initial drug regimen support findings by Asiimwe et al.,^15^ where initiating ART with a TDF-based regimen was associated with better retention than an AZT-based regimen.

One limitation of our study lies in the definition of loss to follow-up, wherein patients were classified as dropped if they did not return to the clinic for 3 mo after their last scheduled visit. Few patients were identified through tracing attempts, partly due to a lack of contact information. If we had used strict definitions for loss to follow-up, such as first gap in care, retention rates would be lower than estimated from other studies, such as the Integrating and Scaling up PMTCT through Implementation Research (INSPIRE) study.^12^ Adopting standardised definitions that translate into health outcomes, as suggested by Rollins et al. (INSPIRE), would inform national HIV programmes.^12^ Challenges existed in collecting and defining patient profiles and ART regimen provision across different data collecting systems. The hub in the north used a different electronic system for data capture from the MOH system than the one introduced at the central hub during the study. Health system challenges such as high staff turnover in the north spokes delayed the updating of ART registers.^9,13,26–29,31^ The investment and efforts needed to support the spokes to sustain and retain patients on treatment, as well as high turnover, remain a challenge, as in other studies.^12^

### Lessons for UTT

In our study, most attrition was due to the high rate of loss to follow-up and efforts to trace individuals revealed undocumented transfers. Outcomes were sporadically recorded on paper and mechanisms to trace patients lost to follow-up were lacking. The limited function of reporting systems for death or tools to measure retention made it difficult to assess outcomes in health facilities without support from implementing partners. Efforts to improve and sustain data collection systems using a hub–spoke mentoring approach to support task-shifting could still be implemented. The latter approach might require more investment in human and financial resources for monitoring.

## Conclusions

Our findings show that adopting a hub–spoke mentoring approach in providing knowledge through training of health workers improved access. Innovations among younger adults and pregnant or breastfeeding women would be valuable for scaling up ART programmes. However, the approach may require investment in improving primary care health facilities, functional monitoring systems and human resource interventions. Further studies assessing the longer-term impact of decentralisation to inform programmes and policies for UTT are warranted.
